# Physiological effects of tourism and associated food provisioning in an endangered iguana

**DOI:** 10.1093/conphys/cot032

**Published:** 2013-11-29

**Authors:** Charles R. Knapp, Kirsten N. Hines, Trevor T. Zachariah, Caro Perez-Heydrich, John B. Iverson, Sandra D. Buckner, Shelley C. Halach, Christine R. Lattin, L. Michael Romero

**Affiliations:** 1 Daniel P. Haerther Center for Conservation and Research, John G. Shedd Aquarium, Chicago, IL, USA; 2 260 Crandon Boulevard, Ste 32 #190, Key Biscayne, FL, USA; 3 Brevard Zoo, Melbourne, FL, USA; 4 Department of Biological Sciences, Meredith College, Raleigh, NC, USA; 5 Department of Biology, Earlham College, Richmond, IN, USA; 6 PO Box N-8893, Villa Capulet, Montague Foreshore, Nassau, The Bahamas; 7 Department of Biology, Tufts University, Medford, MA, USA

**Keywords:** Bahamas, biochemistry, corticosterone, *Cyclura cychlura*, ecotourism, nutrition

## Abstract

Deliberately feeding wildlife is an increasingly popular tourism-related activity despite a limited understanding of long-term impacts on the species being fed. As a result, tourist behaviours that may have adverse impacts on imperiled species have often been encouraged without the necessary evaluation or oversight. Here, we report the responses of Northern Bahamian Rock Iguanas (*Cyclura cychlura*) to human-visitation pressure and associated food provisioning. We compared a variety of blood chemistry parameters of iguanas subjected to supplemental feeding at popular tourist destinations with iguanas occurring on islands where supplemental feeding does not take place. We demonstrate that male and female iguanas inhabiting tourist-visited islands where supplemental feeding occurs do not differ in body condition or baseline stress and stress response (determined by corticosterone levels) compared with iguanas from non-visited islands. Both males and females from tourist-visited sites experienced a greater incidence of endoparasitic infection and atypical loose faeces. Indicators of dietary nutrition, including glucose, potassium, and uric acid values, also differed for both sexes from tourist-visited and unvisited islands. Male iguanas from visited islands differed significantly from those on non-visited islands in calcium, cholesterol, cobalt, copper, magnesium, packed cell volume, selenium, and triglyceride concentrations, whereas female iguanas from visited islands differed significantly in ionized calcium. Although the interpretation of these differences is challenging, chronic biochemical stressors could compromise individual health over time or decrease survivorship during periods of environmental stress. We suggest protocols that can be adopted throughout the region to ensure that supplemental feeding has fewer impacts on these long-lived iguanas.

## Abstract (precis)

Feeding wildlife is an increasingly popular tourism-related activity despite a limited understanding of potential impacts. Here we demonstrate variable differences in physiological values and endoparasitic infection rates between iguanas from tourist-visited and fed populations versus non-visited populations. The responses from iguanas inhabiting visited islands could compromise health over time.

## Introduction

Studies on a variety of wildlife species have demonstrated that tourist activities can influence behaviour (Lott and McCoy, 1995), body condition (Amo *et al.*, 2006), reproductive rates (French *et al.*, 2011), demography (Lord *et al.*, 2001), and physiology (Romero and Wikelski, 2002) of free-ranging animals. Wildlife responses to tourist activities, however, are not always detrimental, suggesting that animals may compensate or habituate to human visitation or that species or age groups may differ in responses to human disturbance (Müllner *et al.*, 2004; Rode *et al.*, 2007; Weinrich and Corbelli, 2009; Hammerschlag *et al.*, 2012).

An increasingly popular yet under-studied tourism-related activity is the deliberate feeding of wildlife. This activity is sanctioned and encouraged for a variety of marine and terrestrial wildlife (e.g. Semeniuk *et al.*, 2009; Hines, 2011; Maljković and Côté, 2011; Foroughirad and Mann, 2013) with minimal understanding of potential health impacts. Food provisioning, especially with unnatural and inappropriate food items, can potentially decrease fitness by providing low-quality or even detrimental dietary additions.

Food provisioning with unnatural, energy-rich diets can lead to obesity and associated deleterious consequences in animals (Warbrick-Smith *et al.*, 2006). Imbalanced nutrition resulting from food provisioning can also cause metabolic syndromes and nutritional disorders (Simpson and Raubenheimer, 2009). Exposure to provisioned food may also alter foraging behaviour and prompt animals to ingest potentially harmful objects (Hines, 2011).

Sources of food provisioning tend to aggregate populations in high densities relative to adjacent natural environments (Oro *et al.*, 2004; Jessop *et al.*, 2012). These fortified aggregations can intensify social conflict and territoriality (Meretsky and Mannan, 1999), skew sex ratios (Jessop *et al.*, 2012), increase parasite/disease transmission (Arneberg *et al.*, 1998), elevate stress levels (Dunkley and Cattet, 2003), weaken immunological responses (French *et al.*, 2010), and ultimately, lead to reduced individual fitness (Jessop *et al.*, 2012).

Food provisioning, however, can also have beneficial effects on individuals that improve fitness, such as increased nutrient intake, improved survival during nutritionally stressed periods, increased growth rates, more robust body condition, augmented fat stores, and enhanced reproductive effort and immunocompetence (Dunkley and Cattet, 2003; Robb *et al.*, 2008; Cotter *et al.*, 2011; Jessop *et al.*, 2012). Interacting with wildlife can also promote health benefits in humans and positive attitudes toward conservation (Orams, 2002; Zeppel, 2008; Ballouard *et al.*, 2012). Thus, the issue of tourism and wildlife feeding is complex, especially in countries heavily dependent on tourism revenues, because the short-term socio-economic benefits are immediate, whereas the potential long-term negative ecological consequences are uncertain (Walpole, 2001).

Tourism companies throughout the Bahamas and Caribbean are increasingly marketing the feeding of endangered rock iguanas (genus *Cyclura*) as part of their activity packages (Knapp, 2004). Northern Bahamian Rock Iguanas (*Cyclura cychlura*) inhabiting the 200-km-long Exuma Island chain in the Bahamas occur naturally on eight cays (small, low islands of limestone and sand) that were historically isolated from heavy visitation pressure. However, Northern Bahamian Rock Iguanas (henceforth referred to as iguanas) have become increasingly popular as feeding attractions. The number of visits to iguana-inhabited cays, with associated feeding, has increased from ∼20 persons per day in the 1980s to currently as many as 150 persons per day on cays visited by tourism operators from Nassau (Iverson *et al.*, 2004a).

**Figure 1.**
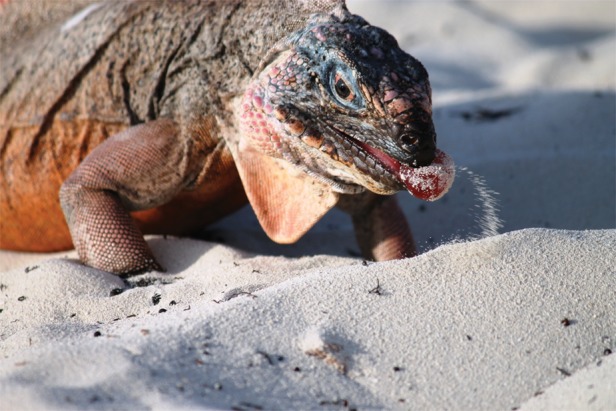
Allen Cays rock iguana from Leaf Cay, Bahamas ingesting a grape fed by a tourist. Photograph courtesy of Kate Hardy.

The economic successes of these tour operators have prompted others to bring hundreds of tourists per week to visit and feed iguanas inhabiting other cays, while at the same time visits from independent tourists are also on the rise. Daily island visits have caused behavioural changes of flight initiation and flight distance (Hines, 2011) and unnaturally high densities at primary landing beaches where iguanas are fed (John Iverson, unpublished data). Despite these behavioural changes, tourism with associated food provisioning in the Bahamas and throughout the Caribbean is likely to increase because of economic benefits. Unfortunately, iguanas are often fed a variety of atypical or inappropriate food items (e.g. bread, cereal, grapes, ground beef, and potato chips) rather than their natural herbivorous diet (Fig. 1). The potential health impacts to target species should be evaluated so that these activities can be managed for the benefit of both wildlife and a sustainable tourism industry. To advance responsible and sustainable management of wildlife tourism involving iguanas worldwide, in the present study we investigate the physiological responses of Bahamian iguanas (*C. cychlura*) to both human visitation and associated artificial food provisioning.

We acknowledge that any physiological response between populations could be either a direct result of visitation and feeding by tourists or an indirect consequence of alterations of nutritional status, increased density, and/or altered social dynamics. We formulated hypotheses based on previous studies and our personal observations. In iguana populations that were visited and fed by tourists, we anticipated, relative to non-visited populations, the following differences: (i) lower body condition indices because of energetic and lost opportunity-to-forage costs of risk avoidance (Frid and Dill, 2002; Amo *et al.*, 2006); (ii) elevated baseline glucocorticoid concentrations and responses because of stress induced by frequent tourist visits and unnaturally high iguana densities on feeding beaches (e.g. Dunkley and Cattet, 2003; French *et al.*, 2010); and (iii) higher endoparasitic rates in iguanas from high-density feeding beaches because transmission rates of parasites are influenced by host densities (Arneberg *et al.*, 1998). We also predicted significant physiological differences in blood chemistry parameters, because these parameters are likely to be influenced by nutritional and environmental conditions (Campbell, 1996).

## Methods

### Focal species and study sites

The Northern Bahamian Rock Iguana is a large-bodied herbivorous lizard (maximum snout–vent length, 62.0 cm; and body mass, 10.38 kg) distributed in isolated populations on islands of the Great Bahama Bank. Of the ∼365 cays in the Exuma Island chain, only eight are currently inhabited naturally by *C. cychlura*, and these populations are separated into two subspecies (*C. c. inornata* and *C. c. figginsi*) that are genetically and morphologically similar (Malone *et al.*, 2003). The iguanas are considered endangered because of their small and fragmented populations, poaching for food and the international pet trade, and introduced mammalian competitors and predators (Blair, 2000; Knapp and Buckner, 2004).

From 28 March to 3 April 2010, we captured iguanas from three cays in the Exuma Island chain that are visited and fed regularly by tourists (Leaf, U, and White Bay Cays) and two cays not visited by tourists (Noddy and North Adderly Cays). All cays are small (mean = 4.7 ha, range = 3–5.9 ha) and characterized by low plant species richness (mean = 36 species; range = 27–54), and similar diversity (mean Sørensen Similarity Index = 0.61; range = 0.53–0.65). Two sites, Leaf and U Cay, are described in detail by Iverson *et al.* (2004b).

### Capture and body condition

We captured sub-adult and adult iguanas on all islands using fish landing nets or by noose. Iguanas were measured for snout–vent length, tail length, and body mass. Gender was determined by cloacal probing for hemipenes. On tourist-visited islands, we captured iguanas on prominent feeding beaches and from isolated, non-visited areas to investigate intra- and inter-island differences in physiological parameters. We calculated body condition indices (Stevenson and Woods, 2006) for each individual as the body mass (in grams)/snout–vent length (in centimetres^3^).

### Baseline stress and response

Immediately following iguana capture, we collected blood samples (4 ml) by venipuncture of the ventral coccygeal vein using a heparinized syringe and stored them in vacutainer tubes containing sodium heparin. We collected all samples within 3 min of capture because corticosterone concentrations, the predominant glucocorticoid present in reptiles (reviewed by Moore and Jessop, 2003), start to increase ∼3 min after the initiation of an acute stressful stimulus in iguanas (Romero and Reed, 2005). We used restraint to investigate the stress response because it is known to elicit large increases in circulating corticosterone levels in reptiles (Romero and Wikelski, 2002; French *et al.*, 2006). We then placed iguanas into an opaque cloth bag and left them alone for 30 min before another blood sample (0.5 ml) was collected using a heparinized syringe to measure stress-related changes in corticosterone (Cort) levels.

### Endoparasites

From 15 to 23 April 2012, we returned to two tourist-visited islands (Leaf and U Cays) and two non-visited islands (Noddy and North Adderly Cays) to investigate the physical condition of faeces (loose and liquid or normal) and endoparasite loads among iguana populations. On visited islands, we restricted our captures to prominent feeding beaches, where iguana densities are artificially inflated because of food provisioning. We collected fresh faecal samples from animals while they were being measured for morphological attributes or from the opaque cloth bags that were used to store iguanas overnight. Samples were immediately scored as normal if the consistency and shape were considered typical, represented in this study as cigar-shaped and moist with evidence of rolled leaves, seeds, or flowers. We scored faecal samples as atypical if they were shapeless, excessively liquid, or consisted only of sand. We stored samples in plastic bags and kept them in a cooler with ice packs until examination for endoparasites. We analysed samples for endoparasitic organisms (adult worms, larvae, ova, oocysts, and cysts) using the zinc sulfate heptahydrate floatation method and via direct smears using standard methodology (Hendrix, 2002).

### Nutritional health

After initial capture and blood sampling during the 2010 field season, we analysed a 0.1 ml subsample of blood immediately in the field using a VetScan i-STAT blood gas analyzer (Abaxis, Union City, CA, USA) with CG8+ cartridges to record ionized calcium, haematocrit, haemoglobin, and pH. We stored the vacutainer tubes on ice (for 4–6 h) until returning to the portable laboratory aboard our research vessel. A small amount of blood was removed and placed into microhaematocrit tubes and centrifuged. We measured packed cell volume using standard methodology, and plasma solids (estimate of proteins) using a refractometer and standard methods (Voigt and Swist, 2011). Plasma was divided into separate aliquots and immediately frozen. We sent one aliquot to Tufts University (MA, USA) for corticosterone analyses by radioimmunoassay after extraction with dichloromethane (Wingfield *et al.*, 1992) with an intra-assay variability of 8%.

We sent a second plasma aliquot to Idexx Laboratories (Elmhurst, IL, USA) to assess standard reptile biochemical parameters, including albumin, calcium, chloride, cholesterol, globulins, glucose, phosphorous, potassium, sodium, total protein, triglycerides, and uric acid. These biochemical parameters are influenced by nutritional status and are commonly used in reptilian diagnostics (Campbell, 1996). Mineral analyses of plasma were conducted at the Diagnostic Center for Population and Animal Health at Michigan State University (East Lansing, MI, USA) and included cobalt, copper, iron, manganese, molybdenum, selenium, and zinc. Although these nutrients are found in trace amounts, they are necessary for proper metabolic function, are obtained by animals through their food, and are a potential indicator of nutritional status (Allen and Oftedal, 2003).

### Statistical analyses

A linear mixed-effects model was fitted to the data to investigate whether differences in blood parameters exist between visited and non-visited sites. Specifically, the model was of the form *Y*_*jk*_ = **X**_*jk*_β + *b*_*j*_ + *e*_*jk*_, where **X**_*jk*_ is a vector of indicator variables for blood parameter *k* within individual *j*, corresponding to sex, visited site status, blood parameter type, and three-way interactions between blood parameter, site, and sex. Individual response variables were transformed to ensure normality of associated error terms and a multivariate normal distribution. Following transformations, all variables were centred and scaled. The term β is a vector of regression parameters, and *b*_*j*_ is a random effect term associated with cay *i*. Sex was included as a covariate to control for potential effects of vitellogenesis because this study was conducted ∼10 weeks prior to oviposition (Iverson *et al.*, 2004b). Hypothesis tests were evaluated via linear contrasts that compared mean blood parameter values between visited and non-visited sites and between male and female iguanas. In order to correct for a potentially inflated false-discovery rate associated with multiple testing, the significance level was adjusted via Benjamini and Hochberg's method (Benjamini and Hochberg, 1995).

A linear mixed-effects model that included sex and visited status as main fixed effects, interaction effects of the two, and a random intercept for site was fitted to determine whether these variables were significantly associated with the change in cortisone levels from baseline to 30 min post-capture, which would indicate a stress response. Furthermore, a series of Student's paired *t* tests, specific to each cay, was conducted to determine whether there was any significant change in cortisone levels within captured individuals. We used Fisher's exact tests to compare faecal consistency and endoparasitic infection rates in animals from visited and non-visited sites. Analyses were conducted in R (R Development Core Team, 2011).

## Results

### Body condition and stress

Iguanas from both visited and non-visited islands did not differ in body condition (Table 1). The change in cortisone levels between baseline and 30 min measurements was not significantly associated with sex or visited site status (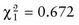, *P* = 0.412; Table 1). However, iguanas on both visited and non-visited islands responded to the stress of capture, handling, and restraint, in that corticosterone concentrations, after 30 min of restraint, were significantly elevated from baseline (*P* < 0.002 across all cays).

**Table 1.** Raw means with ±1 SD of blood chemistry parameters for male and female iguanas captured on visited (1) and non-visited (0) islands

Parameter	Islands		Males		Females
	Visited (1)	*n*	Raw mean (±1 SD)	*n*	Raw mean (±1 SD)
	Non-visited (0)				
Body condition	1	45	0.04 (0.004)	38	0.04 (0.007)
	0	36	0.04 (0.004)	31	0.04 (0.004)
Cort1 (ng/ml)	1	45	5.55 (7.38)	36	8.84 (7.05)
	0	36	4.59 (3.71)	31	7.22 (5.38)
Cort2 (ng/ml)	1	45	15.69 (14.21)	36	19.16 (8.44)
	0	36	14.64 (8.98)	31	20.49 (12.31)
Albumin (g/dl)	1	44	2.02 (0.30)	35	2.08 (0.39)
	0	31	1.97 (0.37)	26	2.07 (0.37)
Calcium (mg/dl)	1	44	10.88 (1.40)	35	16.93 (9.71)
	0	31	9.65 (1.28)	26	18.89 (13.93)
Chloride (mequiv/l)	1	43	121.95 (5.27)	35	121.83 (4.93)
	0	31	125.52 (7.02)	26	122.54 (6.43)
Cholesterol (mg/dl)	1	44	88.66 (37.34)	35	131.37 (70.86)
	0	31	34.58 (17.90)	26	127.81 (100.11)
Cobalt (ng/ml)	1	39	13.55 (7.06)	31	8.84 (4.29)
	0	32	8.10 (5.02)	22	9.90 (4.48)
Copper (ng/ml)	1	39	0.26 (0.119)	31	0.25 (0.098)
	0	32	0.16 (0.072)	22	0.21 (0.061)
Iron (μg/ml)	1	39	62.90 (17.87)	31	56.23 (13.53)
	0	32	63.55 (25.30)	22	48.05 (19.26)
Globulin (g/dl)	1	44	2.85 (0.37)	35	2.94 (0.45)
	0	31	2.89 (0.46)	26	3.08 (0.47)
Glucose (mg/dl)	1	44	150.55 (25.23)	35	150.34 (37.10)
	0	31	120.81 (22.98)	26	111.81 (29.13)
Haematocrit (%)	1	39	22.77 (2.99)	33	21.67 (3.17)
	0	34	21.47 (4.43)	27	21.19 (2.83)
Haemoglobin (g/dl)	1	39	7.73 (1.02)	33	7.36 (1.08)
	0	34	7.29 (1.51)	27	7.20 (0.96)
Ionized calcium (mmol/l)	1	39	1.44 (0.16)	33	1.43 (0.21)
	0	34	1.36 (0.16)	27	1.33 (0.16)
Potassium (mequiv/l)	1	43	2.75 (0.82)	35	2.93 (0.93)
	0	31	3.53 (1.13)	26	3.65 (1.14)
Molybdenum (ng/ml)	1	39	1.79 (2.48)	31	2.97 (4.55)
	0	32	3.06 (4.34)	22	2.20 (2.96)
Manganese (ng/ml)	1	39	0.94 (0.414)	31	3.85 (5.64)
	0	32	0.38 (0.302)	22	7.35 (13.43)
Sodium (mequiv/l)	1	43	169.30 (4.10)	35	166.57 (4.41)
	0	31	169.94 (6.88)	26	167.12 (5.94)
Phosphorus (mg/dl)	1	44	4.47 (1.16)	35	4.83 (1.90)
	0	31	4.32 (1.09)	26	5.57 (2.49)
Packed cell volume (%)	1	44	27.64 (3.57)	35	26.89 (4.34)
	0	35	25.69 (4.62)	30	24.73 (2.64)
pH	1	39	0.14 (0.003)	33	0.14 (0.002)
	0	34	0.14 (0.003)	27	0.14 (0.003)
Selenium (ng/ml)	1	39	57.51 (26.20)	31	46.9 (25.32)
	0	32	36.12 (16.87)	22	42.25 (12.309)
Total protein (g/dl)	1	44	4.87 (0.63)	35	5.02 (0.80)
	0	31	4.86 (0.78)	26	5.15 (0.77)
Triglycerides (mg/dl)	1	44	247.09 (237.50)	35	552.09 (464.28)
	0	31	67.52 (53.22)	26	645.65 (780.78)
Uric acid (mg/dl)	1	44	2.04 (1.55)	35	2.13 (2.16)
	0	31	1.13 (1.52)	26	0.58 (0.84)
Zinc (ng/ml)	1	39	0.69 (0.250)	31	0.78 (0.256)
	0	32	0.63 (0.214)	22	0.94 (0.368)

Cort1 and Cort2 represent baseline and stress-induced corticosterone levels, respectively.

### Endoparasites

Faecal samples collected from iguanas inhabiting visited cays were significantly looser and more liquid (15 of 33) than samples collected from non-visited cays (0 of 13; Fisher's exact test, *P* = 0.004). There was also a higher incidence of the presence of endoparasitic infestation in visited iguana populations (33 of 33 faecal samples) than from non-visited populations (five of 13 faecal samples; Fisher's exact test, *P* < 0.01).

### Nutritional health

**Figure 2.**
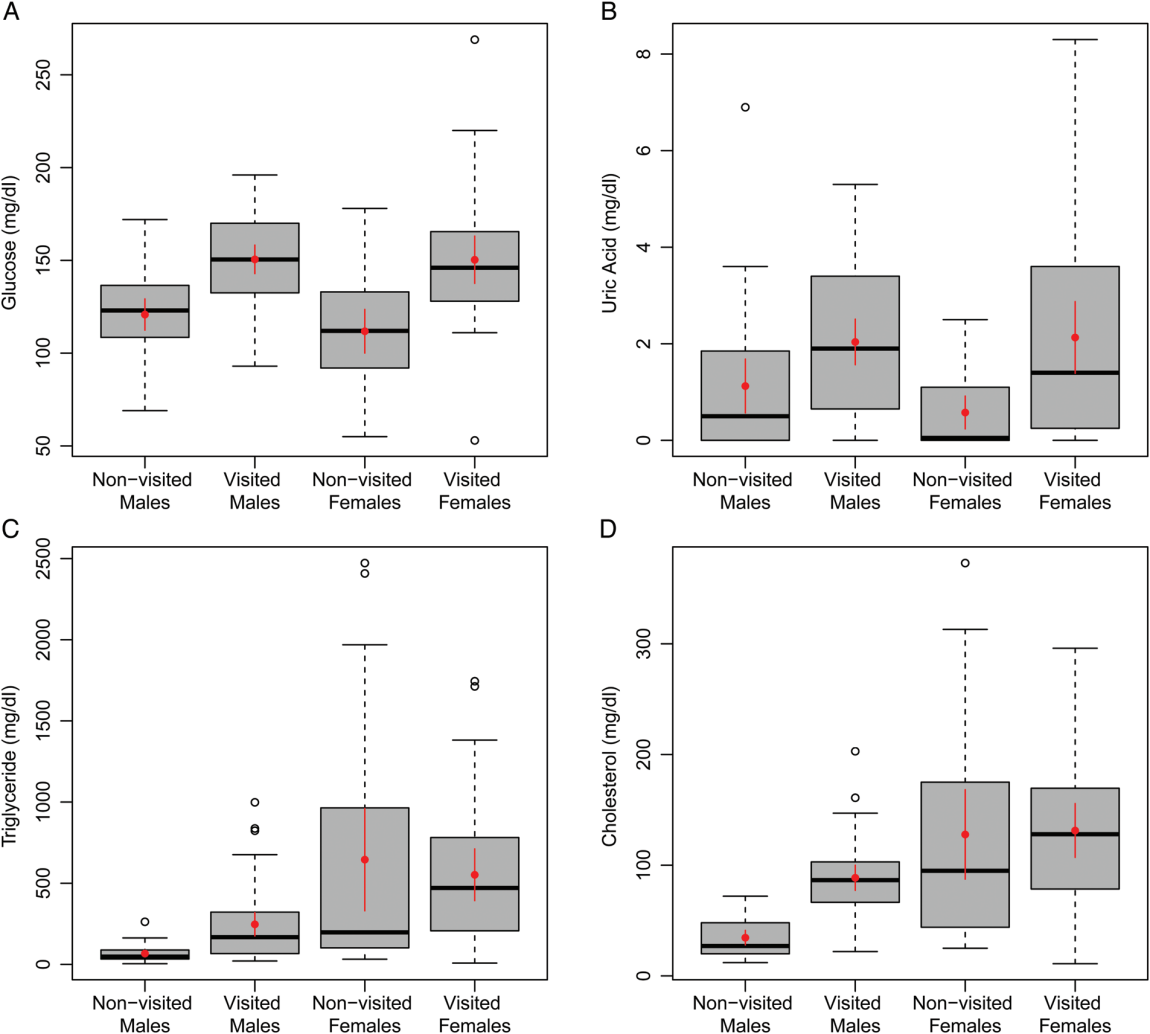
Summary box plots of a subset of blood parameters found to vary significantly by visited vs. non-visited sites. Both sexes differed in glucose (**A**) and uric acid concentrations (**B**). Males only differed in triglyceride (**C**) and cholesterol concentrations (**D**). Data were stratified by sex and visited site status. The dark horizontal bar represents the median, dashed vertical lines indicate variability outside the upper and lower quartiles, and outliers are indicated with open circles. The 95% confidence intervals about the mean (red circle) are displayed in red.

The data set consisted of a total of 25 blood parameters, corresponding to 150 individuals from five islands, for a total sample size of 3478 observations (Table 1). Overall, blood parameters differed significantly between visited and non-visited sites (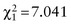, *P* = 0.008). Additionally, an overall test of sex effects indicated that males and females differed significantly with regard to blood parameter values (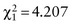, *P* = 0.040). As a result of these sex differences, subsequent results comparing blood chemistry between iguanas at visited and non-visited sites were stratified by sex (Fig. 2). Values for glucose, calcium, magnesium, cholesterol, cobalt, copper, potassium, packed cell volume, triglycerides, uric acid, and selenium differed significantly between males at visited and non-visited sites (Table 2). Values for glucose, ionized calcium, potassium, and uric acid differed significantly between females from visited and non-visited sites (Table 3). No significant differences were detected in biochemical values from iguanas at beach and non-beach sites within visited cays (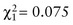, *P* = 0.784).

**Table 2.** Raw mean values with standard deviations, test statistics, and *P*-values for physiological parameters that differed significantly between male iguanas at visited and non-visited sites

Parameter	Raw mean (SD)	Raw mean (SD)	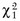	*P*-value
	Non-visited sites	Visited sites		
Calcium (mg/dl)	9.65 (1.28)	10.88 (1.40)	3.950	0.047
Cholesterol (mg/dl)	34.58 (17.90)	88.66 (37.34)	30.842	<0.001
Cobalt (ng/ml)	8.10 (5.02)	13.55 (7.06)	21.278	<0.001
Copper (ng/ml)	0.16 (0.072)	0.26 (0.119)	17.262	<0.001
Glucose (mg/dl)	120.81 (22.98)	150.55 (25.23)	15.905	<0.001
Potassium (mequiv/l)	3.53 (1.13)	2.75 (0.82)	9.361	0.002
Manganese (ng/ml)	0.38 (0.302)	0.94 (0.414)	5.357	0.021
Packed cell volume (%)	25.69 (4.62)	27.64 (3.57)	6.287	0.012
Selenium (ng/ml)	36.12 (16.87)	57.51 (26.20)	15.148	<0.001
Triglycerides (mg/dl)	67.52 (53.22)	247.09 (237.50)	13.130	<0.001
Uric acid (mg/dl)	1.13 (1.52)	2.04 (1.55)	7.809	0.005

Significance levels were adjusted for multiple comparisons using the Benjamini and Hochberg (1995) approach.

**Table 3.** Raw mean values with standard deviations, test statistics, and *P*-values for physiological parameters that differed significantly between female iguanas at visited and non-visited sites

Parameter	Raw mean (SD)	Raw mean (SD)	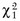	*P*-value
	Non-visited sites	Visited sites		
Glucose (mg/dl)	111.81 (29.13)	150.34 (37.10)	22.951	<0.001
Ionized calcium (mmol/l)	1.33 (0.16)	1.43 (0.21)	5.011	0.025
Potassium (mequiv/l)	3.65 (1.14)	2.93 (0.93)	6.349	0.012
Uric acid (mg/dl)	0.58 (0.84)	2.13 (2.16)	13.719	<0.001

Significance levels were adjusted for multiple comparisons using the Benjamini and Hochberg (1995) approach.

## Discussion

### Body condition

Body condition is linked to fitness (Viblanc *et al.*, 2012) and has been used to quantify the health of lizards in tourist-visited areas (Amo *et al.*, 2006). Reacting to tourist disturbance and food provisioning can negatively or positively affect body condition via the energetic and lost opportunity-to-forage costs of risk avoidance (Frid and Dill, 2002) or via increased nutritional inputs (Jessop *et al.*, 2012), respectively. Iguanas from feeding beaches of visited islands occur at greater densities, do not perceive tourists as threats, and often approach tourists (Hines, 2011). Although targeted, long-term studies are required to isolate the mechanistic causes for similar body condition between islands, we suspect that the lack of avoidance behaviour may mitigate any lost opportunity-to-forage costs, while increased iguana densities on visited islands may negate positive responses to increased dietary additions.

### Corticosterone concentrations

Although we suspected that higher iguana densities on visited beaches and high-frequency visits by tourists would result in elevated corticosterone values, levels did not differ significantly between visited and non-visited iguana populations. These data suggest that iguanas on islands visited by tourists are not chronically stressed. Iguanas, however, exhibited elevated corticosterone levels after 30 min of capture and restraint, indicating that both groups can respond physiologically to stressful stimuli. Our results are unusual because most tourism-related studies using corticosterone as a metric for anthropogenic disturbance have found either lower (Fowler, 1999) or higher (Ellenberg *et al.*, 2007; Thiel *et al.*, 2008) baseline levels of corticosterone in visited areas or differences in the corticosterone response between tourist and non-tourist areas after a stressor (Romero and Wikelski, 2002; Müllner *et al.*, 2004; French *et al.*, 2010). Previous studies, however, have not examined tourist-visited populations that were also fed.

The iguana species in this study does not demonstrate conventional territorial behaviour as reported in other species. Instead, it is suspected that relative high densities are responsible for territorial to hierarchical behavioural shifts in *C. cychlura* populations inhabiting small islands (Knapp, 2000). Consequently, these animals appear to have adjusted and responded behaviourally to high densities prior to the onset of tourism and food provisioning. Additionally, iguanas from populations in our study exposed to prolonged visitation and food provisioning apparently do not view humans as a threat, probably because of their positive association with food. Once habituated, these iguanas have significantly shorter flight initiation and flight response distances (Hines, 2011), and often approach humans as a source of food rather than perceiving them as a potential threat. Equivalent corticosterone responses in these visited and non-visited populations fit the proposed criteria for hormonal habituation in wildlife (Cyr and Romero, 2009). Unfortunately, the lack of a threat response to humans may be detrimental to their survival in the long run, because it facilitates ease of capture for illegal wildlife smugglers or poaching for human consumption (IRCF, 2009), as well as unsanctioned and illegal relocation by tour operators when they are deemed too aggressive for tourists (Smith and Iverson, 2006).

### Endoparasites

In this study, the incidence of endoparasitic infestation was greater in visited populations than in non-visited populations. Free-ranging reptiles are infested naturally with a great diversity of endoparasites, yet relatively few reports link parasite burdens with morbidity or mortality events in wild reptiles. Indeed, in most cases, the reptilian host–parasite relationship has yet to be fully understood and documented (Jacobson, 2007). The majority of endoparasites identified in this study were the ova from *Ancylostoma* spp. (hookworms) and *Oxyurus* spp. (pinworms). The oxyurids are considered commensal and generally regarded to be non-pathogenic in iguanas (James *et al.*, 2006), although heavy burdens can lead to clinical disease (Diaz-Figueroa and Mitchell, 2006). Oxyurids can achieve significant population numbers within the colon, especially in herbivorous iguanids (Iverson, 1982), which may put visited populations at greater risk for impaction (Kane *et al.*, 1976). The risk for impaction may also be greater for visited populations because sand is often ingested non-selectively when encrusted on wet grapes and other fed items (Fig. 1; Hines, 2011).

The 100% endoparasitic infection rate and higher endoparasitic loads for iguanas from visited islands suggest that unnaturally high iguana densities resulting from supplemental feeding may pose a larger health risk through the transmission of parasites and possible disease. The combination of high density and overlap with humans may also make these populations susceptible to reverse zoonotic diseases spread by humans (Wheeler *et al.*, 2012). Future microbiology and parasitology analyses are warranted to improve our understanding of how human associations influence disease risk in iguanas and other wildlife.

### Nutritional health

Both sexes on visited cays consume food distributed by tourists, although male iguanas are more aggressive when feeding and eat more provisioned food (Iverson *et al.*, 2004a). Consequently, they may be more impacted by provisioning with unnatural foods, as suggested by the greater suite of significant physiological differences in males between populations (Table 2).

Plasma glucose concentrations in iguanas of both sexes inhabiting tourist-visited islands were significantly higher than in iguanas inhabiting non-visited islands (Tables 2 and 3). We suspect that the increased glucose values are the result of being fed high concentrations of sugary fruits (e.g. grapes) daily (Hines, 2011; John Iverson, personal observation). An over-abundance of grapes in the diets of iguanas from tourist-visited cays may also be responsible for the excessive diarrhoea observed on feeding beaches and quantified in this study.

Plasma potassium levels for both sexes were significantly lower in iguana populations visited and fed by tourists (Tables 2 and 3). The significantly lower potassium concentrations in visited populations most probably result from inadequate dietary intake of potassium or excessive potassium loss through diarrhoea (Campbell, 1996), the latter of which is often observed on feeding beaches and quantified in this study. Iguanas on visited islands predominately eat grapes that are provided by tour operators on a daily (weather-dependent) basis. Grapes are inherently low in potassium (USDA Nutrient Data Laboratory, 2011) and possess 3–10 times less potassium than 10 of the most common native plants occurring on the islands (Charles Knapp and Silvia Alvarez Clare, unpublished data).

The major nitrogenous waste product of protein catabolism in reptiles is uric acid (Campbell, 1996). Chronically increased plasma uric acid concentrations in both sexes from visited islands may indicate a susceptibility to renal disease and predispose animals to gout. Males from visited islands in this study exhibited increased cholesterol concentrations. Although cholesterol is a useful diagnostic parameter in mammalian biochemistry, the relationship between cholesterol levels and disease is not fully understood in reptiles. However, herbivorous wildlife in general should have stable cholesterol, and thus unusual increases may indicate the introduction of meat to their diets (personal communication from Bonnie Raphael, Wildlife Conservation Society).

The higher uric acid levels in male and female iguanas on visited islands and elevated cholesterol levels in males from visited islands could be the result of animal protein (e.g. ground beef) being fed to iguanas by tourists (Smith and Iverson, 2006). Additionally, food provisioning by tourists on beaches has encouraged iguanas to spend disproportionate amounts of time foraging at the wrack line instead of the island interior (Hines, 2011). Consequently, iguanas may ingest higher levels of marine-sourced protein (e.g. invertebrates or fish) washed ashore on visited islands.

Along with cholesterol, triglyceride concentrations are among the most conspicuous physiological differences between male iguanas from visited and non-visited islands (Table 1). Triglyceride concentrations for males from visited islands in this study are beyond reported reference intervals for other *Cyclura* (Lung, 2012). As in humans (Miller *et al.*, 2011), diet can affect triglyceride levels in reptiles (Cartland *et al.*, 1994; Barboza *et al.*, 2011), especially with foods containing excessive sugar and starch, both of which are fed excessively to iguanas on visited cays. Examples include grapes, cereal, white bread, and potato chips (Hines, 2011; Charles Knapp, personal observation). To our knowledge, direct health impacts have yet to be investigated for reptiles experiencing abnormally high triglyceride levels. Nevertheless, triglyceride concentrations should remain a parameter of interest for future investigations.

Although it is assumed that trace elements constitute an important component of the reptilian diet (Allen and Oftedal, 2003), the effects of mineral status on wild reptiles are poorly understood. We recorded differences in trace mineral values in males (cobalt, copper, manganese, and selenium) from visited and non-visited islands. We postulate that dietary differences (i.e. artificial vs. natural) between visited and non-visited populations may influence mineral values in our study because elements of the diet can reduce (e.g. phytate, oxalate, and calcium) or enhance (e.g. vitamin C) trace element absorption from the digestive system (Allen and Oftedal, 2003). Alternatively, the differences are possibly the result of mineral uptake from ingested wet grapes encrusted with sand or ingested marine-sourced protein in the wrack line. Iguanas regularly ingest sand when fed grapes (Fig. 1), and significantly more faecal samples are impacted with sand on visited vs. non-visited islands (Hines, 2011). The sand is tidally inundated with seawater or exposed to constant salt spray, potentially elevating the uptake of trace minerals artificially through non-selective geophagy. The non-selective ingestion of sand may also lead to greater health risks due to impaction, as recorded previously in visited populations only (Hines *et al.*, 2010; Iverson *et al.*, 2011).

We failed to identify a statistical difference in physiological parameters between primary landing beaches and more remote areas of islands visited by tourists. A previous study did reveal differences in uric acid and glucose concentrations from Leaf Cay between habituated iguanas on the beach and non-beach individuals (James *et al.*, 2006). We suspect that the lack of statistical significance in our study is due to smaller sample sizes (Leaf, non-beach 17 and beach 15; U-Cay, non-beach 11 and beach 14; and White Bay, non-beach 12 and beach 14) or that all iguanas inhabiting the island may have habituated to visitation over time and are now moving between feeding beaches and non-visited areas more frequently.

Interpretation of reptilian clinical chemistry values has not yet achieved the level of precision realized in small mammals and birds (Campbell, 1996). Nevertheless, we have demonstrated that biochemical concentrations are most probably influenced by food provisioning from tourists. Although many of the biochemical plasma values in this study fall within published reference values for *Cyclura* (Lung, 2012), normal blood chemistry values may vary significantly among different reptile species (Campbell, 1996). We stress the importance of this intra-specific comparison instead of solely evaluating published references. The biological effects of altered biochemical concentrations may not be manifested over a short time period, but could have deleterious impacts on long-term fitness and population stability. For example, food provisioning of garden birds can have important downstream consequences, such as smaller relative yolk mass in larger eggs and reduced yolk carotenoid concentrations in early breeders (Plummer *et al.*, 2013). The long-term consequences of tourism and food provisioning for iguanas are uncertain, and thus studies targeting measurements of fitness (e.g. reproductive success, survival) are encouraged. Indeed, the iguanas in this study are long lived (>50 years; Iverson *et al.*, 2004b), and thus chronic biochemical stressors could compromise health and population stability over time, or reduce the ability to survive during periods of adverse environmental stress. Minimally, these biochemical data should be used as a baseline for further research and as a reference for advancing sustainable management of wildlife tourism throughout the region.

### Recommendations

Iguana species are distributed throughout the Neotropics and are conspicuous symbols for the countries and environments they inhabit. Iguanas, however, receive less management attention than other charismatic fauna. Results from this study and the behavioural evaluations of Hines (2011) provide a quantitative conclusion that unregulated food provisioning alters the physiology and behaviour, and facilitates higher endoparasitic infection rates of the Northern Bahamian Rock Iguana. However, we acknowledge the simplistic assumption that any alteration is negative, because it ignores the evidence that feeding can have beneficial responses. Increased population density can be viewed as a positive response, especially for an endangered species. Unnaturally high densities and an excessive reliance on tourists for food, however, may prove disadvantageous if food supplementation is discontinued for extensive periods of time. Plant community dynamics can also be disrupted if iguanas cease foraging naturally in favour of human-provided foods. West Indian iguanas, as the dominant native herbivores on islands where they occur, play a vital ecological role by promoting foliage growth through cropping, providing nutrients to developing seedlings, and dispersing seeds to new areas (Auffenberg, 1982; Iverson, 1985). Furthermore, the lack of a threat response makes the animals more susceptible to capture for the illicit wildlife trade or human consumption, as well as illegal relocation when they are deemed too aggressive for tourists.

While some natural resource managers may agree that the endangered status of these iguanas should supersede recreational tourism, we do not endorse reducing or modifying tourist visitation at this time. Indeed, we recognize the economic and potential educational benefits of this type of activity. With modifications, for example, a similar food-based tourism enterprise for iguanas in the Turks and Caicos Islands was successfully transformed into a non-feeding educational tour along boardwalks (Hines, 2011).

The complete restriction of feeding may not be a realistic option. Instead, wildlife managers could approach manufacturers of pelleted iguana foods and request a specially formulated food to mitigate the impact of provisioning with unhealthy food. Tour operators could offer or sell such pellets to their clients, which would provide a more nutritionally balanced diet and reduce non-selective ingestion of sand encrusted on wet fruit. Delivery systems could also be modified to reduce ingestion of sand. One tour company now encourages tourists to feed grapes impaled on the ends of sticks. Although the food item is still an issue, sand ingestion is reduced considerably. Other options include limiting the number of cays where iguanas can be visited and fed. It is important, however, that tourists are prohibited from visiting certain cays to keep iguana populations in a natural state so that they may serve as controls for interpreting long-term physiological and demographic impacts. We also endorse a broad education campaign and discourage references to feeding iguanas in advertisements. Minimally, we urge serious discussions among wildlife managers and stakeholders to identify tactics that mitigate the impacts of current tourism practices without compromising an important economic activity.
